# CDC37 as a novel target for the treatment of NPM1-ALK expressing anaplastic large cell lymphomas

**DOI:** 10.1038/s41408-019-0171-2

**Published:** 2019-01-29

**Authors:** Sudhakiranmayi Kuravi, Elizabeth Parrott, Giridhar Mudduluru, Janice Cheng, Siddhartha Ganguly, Yogen Saunthararajah, Roy A. Jensen, Brian S. Blagg, Joseph P. McGuirk, Ramesh Balusu

**Affiliations:** 1 Division of Hematologic Malignancies and Cellular Therapeutics, Department of Internal Medicine, University of Kansas Medical Center, Kansas City, KS USA; 2 University of Kansas Medical School, Kansas City, KS USA; 3 Department of Translational Hematology and Oncology, Taussig Cancer Institute, Cleveland Clinic, Cleveland, OH USA; 4 Department of Pathology and Laboratory Medicine, University of Kansas Medical Center, Kansas City, KS USA; 5 Department of Chemistry and Biochemistry, University of Notre Dame, Notre Dame, IN USA; 6 Division of Hematologic Malignancies and Cellular Therapeutics, Department of Internal Medicine, University of Kansas Medical Center, Kansas City, KS USA; 7 Department of Cancer Biology, University of Kansas Medical Center, Kansas City, KS USA

Anaplastic large cell lymphoma (ALCL) represents a rare and aggressive subtype of CD30-positive peripheral T-cell lymphoma, which accounts for 5–10% of non-Hodgkin lymphomas in adults and 10–30% in children^1^. Anaplastic lymphoma kinase (ALK) fusions are present in both solid and hematologic malignancies. More than 80% of ALK-positive ALCLs are hallmarked by the fusion gene nucleophosmin (NPM1)-ALK generated by the *t*(2;5) chromosomal translocation, and 5% of non-small cell lung cancer patients carry echinoderm microtubule-associated protein-like 4 (EML4)-ALK fusion^1^. NPM1 is a nucleolar phosphoprotein involved with chaperoning of proteins and nucleic acids^2^. ALK is a receptor tyrosine kinase belonging to the insulin receptor superfamily. NPM1-ALK fusion protein p80 is derived from the fusion of the N-terminal oligomerization domain of NPM1 (1–110 aa) and the C-terminal tyrosine kinase domain of ALK (1058–1620 aa)^3^. In these malignancies, the homo-dimerization of NPM1-ALK leads the constitutive activation of a fusion kinase in a ligand-independent manner. The persistently active tyrosine kinase NPM1-ALK triggers multiple intracellular downstream signaling pathways including AKT, ERK1/2, and STAT3, which results in proliferation and survival of ALCL cells^1^.

Many ALK tyrosine kinase inhibitors have been developed, evaluated, and approved for clinical trials in malignancies associated with ALK fusion genes. Clinical resistance is a major problem associated with these ALK inhibitors. One of the explored mechanisms responsible for resistance to ALK inhibitors in these malignant cells is through acquired secondary mutations in the kinase domain^4^. Overall, current therapeutic approaches used for the treatment of ALK-positive ALCLs has limited effectiveness, resulting in a substantial percentage of cases with poor outcomes, either failing to achieve remission or relapsing within a short period. Hence, there is a need to focus on developing novel and effective treatment strategies to overcome this clinical conundrum.

More than 500 protein kinases are present in the human kinome and they play an essential role in cellular processes such as cell proliferation, signaling, differentiation, and apoptosis^5^. Dysregulation of these protein kinase functions (genetic alterations including mutations or fusion genes) is responsible for various pathological conditions including cancer. Protein kinases depend on a central molecular chaperone, heat shock protein 90 (HSP90) for their maturation and to protect against proteasomal degradation. HSP90 is an ATPase-dependent master chaperone that accounts for around 10% of the proteome. It is important to note, the majority of mutated kinase proteins involved with malignancies are maintained through HSP90 dependency, leading to “HSP90 addiction.” Exploitation of HSP90 by oncogenic kinases for their stability and maturation makes HSP90 as a viable molecular target^6^. Several HSP90 inhibitors bind to the ATP binding site of HSP90 and promote degradation of oncogenic protein kinases. Mutant fusion kinases such as BCR-ABL and NPM1-ALK are known HSP90 client proteins in hematologic malignancies^7^. Several preclinical studies have been very supportive in considering HSP90 as a therapeutic target. However, many clinical trials using HSP90 inhibitors have been halted due to toxicity associated with the robust heat shock response^8^. An alternative approach to effectively target these HSP90-dependent oncogenic kinases, while minimizing non-specific toxicity could be disrupting the interaction between HSP90 and its co-chaperones. HSP90 chaperone machinery is complex, and along with HSP90, many co-chaperones are involved in the process. Cyclin-dependent kinase 37 (CDC37) is a specific co-chaperone recruiter for a diverse group of protein kinases. HSP90-mediated maturation of these oncogenic kinases strictly depends on CDC37^9^. Celastrol, a triterpene molecule extracted from the Chinese herbal plant *Tripterygium wilfordii* Hook F, blocks the interaction between HSP90 and CDC37 and thereby triggers the degradation of its dependent client protein kinases. Mechanistically, celastrol does not interfere with ATP binding to HSP90, but inhibits the critical interaction and binding between the N-terminal region of CDC37 (Arg167) and the middle domain of HSP90 (Glu33)^10^.

In this study, we tested the fusion oncogene NPM1-ALK dependency on co-chaperone CDC37 by disrupting the interaction between HSP90 and CDC37, using celastrol as a therapeutic approach in ALCL cells. We have utilized a total of six cell lines: NPM1-ALK endogenously expressing human ALCL cell lines (SUDHL-1, Karpas-299, SUP-M2, SR-786, and DEL), and our laboratory generated ectopically overexpressing Ba/F3-FG-NPM1-ALK, a murine cell line. In this report, we present celastrol-mediated effects on apoptosis, proliferation, oncogenic signaling, and CD30 (cluster of differentiation 30) expression in ALCL cells.

Earlier studies demonstrated NPM1-ALK as an HSP90 client protein by using the HSP90 ATPase inhibitor-17AAG^11^. Several protein kinases are well characterized for their dependency on CDC37 co-chaperoning, but very limited studies are available for fusion kinases. In hematologic malignancies, BCR-ABL was the first fusion kinase identified to be dependent on CDC37 co-chaperone interaction for its stability^12^. Our experiments confirmed that endogenous NPM1-ALK fusion protein levels in SUDHL-1, Karpas-299, SUP-M2, SR-786, and DEL cells were diminished with celastrol treatment in a dose-dependent manner (0.25–1.0 µM) after 24 h (Fig. 1a). In similar lines, ectopically overexpressed NPM1-ALK was also downregulated in Ba/F3 cell line. The decrease in total NPM1-ALK resulted in a reduction of active phosphorylated NPM1-ALK (Fig. 1a). With the significant decrease in protein levels of total NPM1-ALK and phospho-NPM1-ALK, we further examined the influence of these effects on relevant NPM1-ALK downstream signaling in five NPM1-ALK expressing cell lines. AKT/PI3K, MAPK/ERK, and STAT3 are well-studied survival signaling pathways that are activated by NPM1-ALK in CD30-positive ALCL cells^13^. Celastrol-mediated downregulation of NPM1-ALK phosphorylation inhibited downstream signaling activators phosphorylated AKT, ERK1/2, and STAT3 in a dose-dependent manner. There was a minimal effect on total AKT, ERK1/2, STAT3 proteins, and β-actin levels were used as loading control (Fig. 1b). Based on these experimental results, celastrol downregulates fusion protein NPM1-ALK by blocking the interaction between HSP90 and CDC37, which in turn inhibits downstream survival signaling cascade AKT, ERK1/2, and STAT3.

**Fig. 1:**
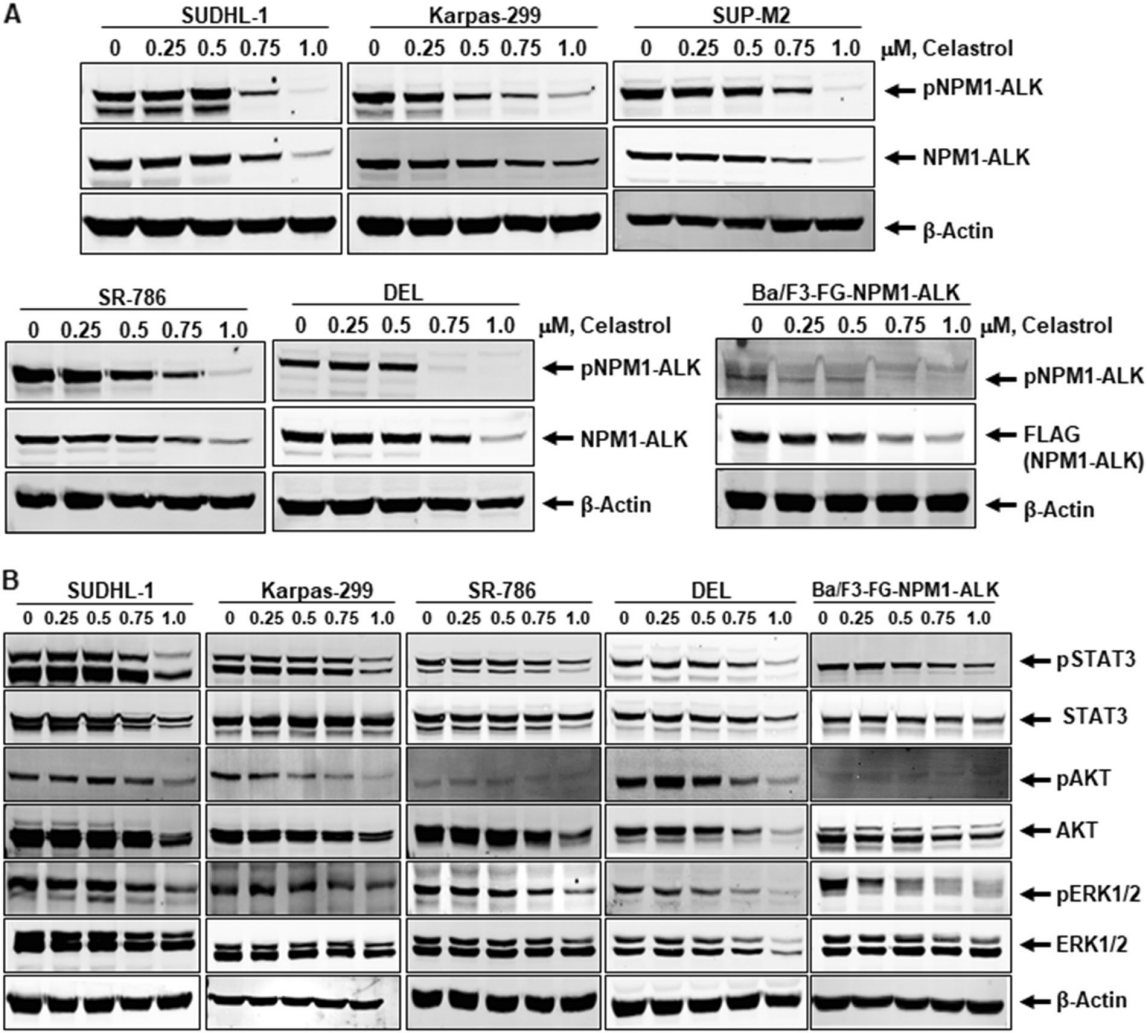
Celastrol downregulates NPM1-ALK fusion protein and its signaling: (**a**) celastrol treatment depletes NPM1-ALK protein levels and inhibits activation of NPM1-ALK fusion kinase. NPM1-ALK expressing SUDHL-1, Karpas-299, SUP-M2, SR-786, DEL, and Ba/F3-FG-NPM1-ALK cells were treated with indicated concentrations of celastrol for 24 h. At the end of the treatment period, cell lysates were made, and immunoblot analyses were performed for total NPM1-ALK and phospho-NPM1-ALK proteins. **b** Depletion of NPM1-ALK leads to inhibition of downstream survival signaling cascade. NPM1-ALK expressing ALCL cell lines treated with celastrol and western blot analyses were performed for downstream effector molecules pSTAT3, pAKT, pERK1/2 along with total proteins. β-actin served as the loading control

We then evaluated the ability of celastrol to induce apoptosis in NPM1-ALK endogenously expressing SUDHL-1, Karpas-299, SUP-M2, SR-786, DEL, and ectopically expressing Ba/F3-FG-NPM1-ALK lymphoma cell lines along with normal T cells. All selected ALCL cell lines were treated with celastrol (0.1–1.0 µM) for 48 h, and apoptosis was measured by flow cytometry using FITC-annexin V and TO-PRO-3. All of the tested NPM1-ALK fusion gene expressing cell lines were sensitive to celastrol and showed induced apoptosis in a dose-dependent manner compared to controls but no significant effect on normal T cells (Fig. 2a). Overall, celastrol showed growth inhibitory effects on both endogenous and ectopic NPM1-ALK expressing cell lines. PARP (poly (ADP-ribose) polymerase) catalyzes poly(ADP-ribosyl)ation of nuclear proteins involved in DNA transcription, replication, and repair. During apoptosis, it is well known that PARP is cleaved by specific caspases. Cancer cells are associated with an imbalance between pro- and anti-apoptotic genes^14^. Therefore, we examined PARP cleavage, activation of caspases, and differential regulation of pro-apoptotic (BAX) and anti-apoptotic molecules (survivin, Bcl2, and c-Myc). Two cell lines, SUDHL-1 and Karpas-299 were treated with 0.25–1.0 µM celastrol for 24 h. Celastrol treatment in these cell lines showed PARP cleavage, downregulation of procaspases 8 and 9, upregulation of pro-apoptotic protein BAX and downregulation of anti-apoptotic proteins survivin, Bcl2, and c-Myc in a dose-dependent manner (Fig. 2b). All the evaluated proteins involved in apoptosis are activated by the downstream signaling axis of NPM1-ALK^13^.

**Fig. 2:**
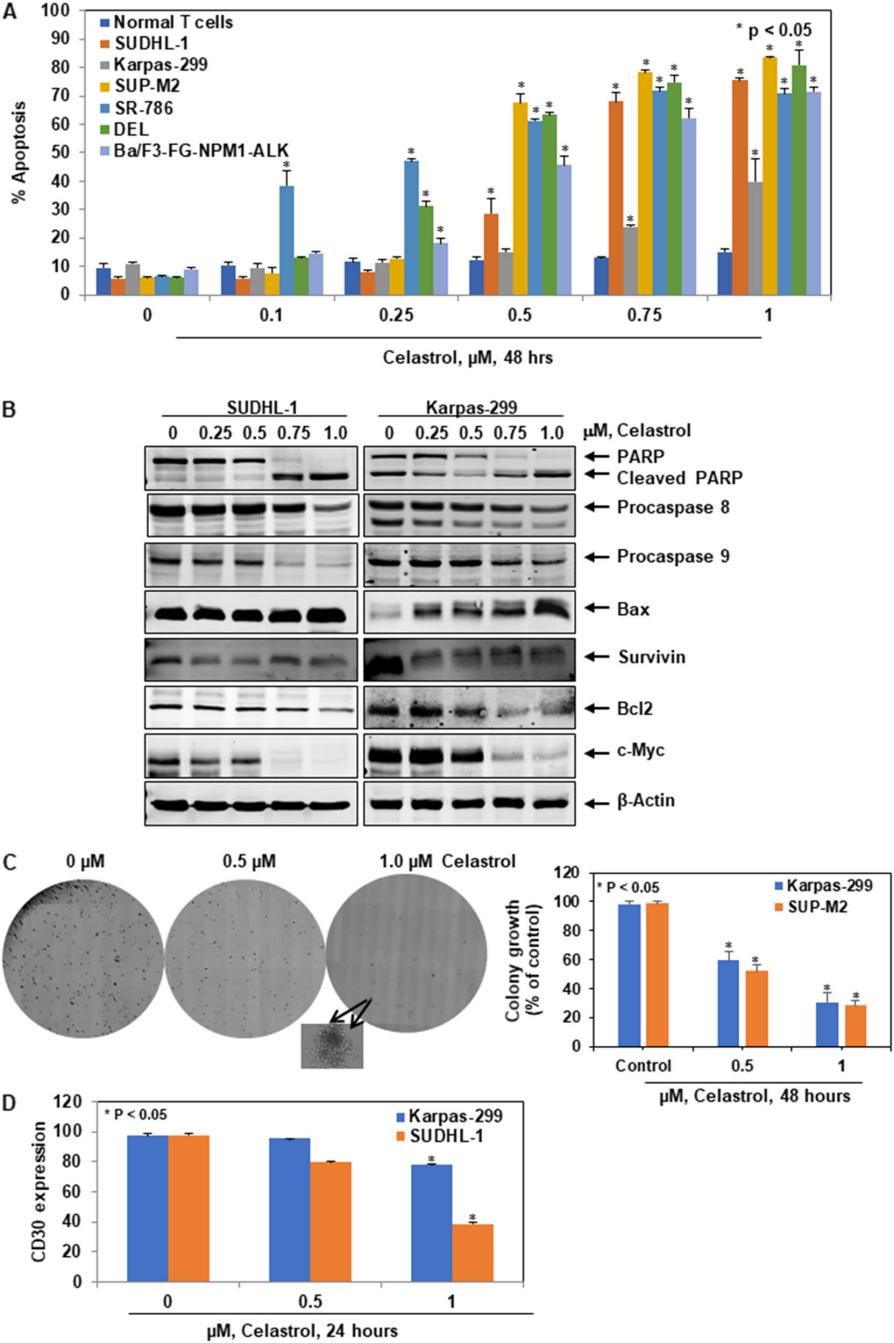
Celastrol induces apoptosis in ALCL cells: (**a**) downregulation of NPM1-ALK induces apoptosis. The NPM1-ALK expressing SUDHL-1, Karpas-299, SUP-M2, SR-786, DEL, Ba/F3-FG-NPM1-ALK cell lines and normal T cells (*n* = 3) were treated with the indicated concentrations for 48 h. After treatment, cells were harvested and stained with FITC-annexin V and TO-PRO3, and the percentages of apoptotic cells were determined by flow cytometry. Columns represent the mean of three independent experiments; bars represent the standard error of the mean (SEM). **b** Effect of celastrol on apoptotic proteins: SUDHL-1 and Karpas-299 ALCL cell lines were treated with the indicated concentrations of celastrol for 24 h. At the end of the treatment, cell lysates were prepared and western blot analyses were performed for cleaved PARP, procaspases 8, 9, Bax, survivin, Bcl2, and c-Myc. β-Actin served as loading control. **c** Celastrol inhibits the clonogenic potential of ALCL cells. Karpas-299 and SUP-M2 cells were treated with indicated concentrations of celastrol for 24 h, washed, mixed with MethoCult medium, and plated. After 8 days of incubation, the number of colonies were counted. A portion of the full image is enlarged for clear visualization of colonies (Karpas-299). Columns represent the mean of three independent experiments; bars represent the SEM. **d** Celastrol downregulates the CD30 surface expression in ALCL cell lines. The cells were treated for 24 h with indicated concentrations of celastrol and stained with anti-CD30-FITC along with respective isotype control antibodies. The CD30-positive cells were measured by flow cytometry. Columns represent the mean of three independent experiments; bars represent the SEM

The chimera NPM1-ALK drives the proliferation of ALCL cells. Therefore, we tested the effect of celastrol on the proliferation of NPM1-ALK-positive cells using a standard methylcellulose clonogenic assay. Karpas-299 and SUP-M2 cells were treated with 0, 0.5, and 1.0 µM celastrol for 24 h. Cells were then washed, mixed with a MethoCult medium, plated, and incubated for 8 days. The total number of colonies were counted in each condition. There was a significant reduction in the clonogenic potential of celastrol-treated Karpas-299 and SUP-M2 cells compared to control due to inhibition of NPM1-ALK activation (Fig. 2c).

ALCL cells are immunophenotypically characterized by the strong expression of the CD30 marker, a member of the tumor necrosis factor (TNF) receptor family, which is transcriptionally upregulated through the NPM1-ALK-mediated ERK1/2 and STAT3 pathways^15^. CD30 activation contributes to lymphoma cell proliferation through activated NF-κB and other anti-apoptotic mechanisms. We analyzed the effect of NPM1-ALK downregulation on CD30 expression in Karpas-299 and SUDHL-1 cells (Fig. 2d). ALCL cell lines were treated with celastrol and analyzed for CD30 expression by flow cytometry. The results showed treatment with celastrol in the SUDHL-1 cell line exhibited a higher response in the reduction of CD30 in comparison to Karpas-299 cell line. The differential response might be due to the variable expression levels of NPM1-ALK and downstream signaling effector molecules in these cell lines.

In summary, our results show for the first time that inhibition of the interaction between CDC37 and HSP90 using celastrol represents a novel therapeutic approach in ALCL cells expressing the NPM1-ALK fusion gene. These observations further pave the way to consider CDC37 as a novel molecular target for the treatment of NPM1-ALK expressing ALCL cells and warrants developing future therapeutic intervention strategies.

## Supplementary information

Supplementary Material
