# Supplementary material for: CDC37 as a novel target for the treatment of NPM1-ALK expressing anaplastic large cell lymphomas
Source: Blood Cancer J. 2019 Jan 29;9(2):14. doi: 10.1038/s41408-019-0171-2 (PMC6351628; doi:10.1038/s41408-019-0171-2)
Supplement: Supplementary file 1 — Supplementary Material [file 41408_2019_171_MOESM1_ESM.docx]

**CDC37 As a Novel Target for the Treatment of NPM1-ALK Expressing Anaplastic Large Cell Lymphomas**

Sudhakiranmayi Kuravi, Elizabeth Parrott, Giridhar Mudduluru, Janice Cheng, Siddhartha Ganguly, Yogen Saunthararajah, Roy A Jensen, Brian S Blagg, Joseph P McGuirk, Ramesh Balusu

**Supplementary Material**

**Cell culture:** SUDHL-1, DEL, SR-786, SUP-M2, Ba/F3 cell lines were obtained from DSMZ (Deutsche Sammlung von Mikroorganismen und Zellkulturen GmbH, Braunschweig, Germany) and WEHI-3B, Karpas-299 cell lines from ATCC (American Type Culture Collection Manassas, VA, USA). WEHI-3B cells were grown in DMEM medium and remaining all other cell lines were maintained in RPMI-1640 medium supplemented with 10% FBS, and 1% penicillin/streptomycin (Thermo Scientific, Waltham, MA, USA) and Ba/F3 cells were maintained in RPMI-1640 medium supplemented with 10% FBS, 1 % penicillin/streptomycin, 10% WEHI-3 conditioned medium (as a source of interleukin 3 - IL3), and 2 µg/ml of puromycin**.**

**Isolation of normal T cells:** Healthy individual blood samples were procured from Biorepository Core Facility at University of Kansas Medical Center, Kansas City, KS. Normal T cells were isolated from the blood samples using EasySep human T cell isolation kit from StemCell Technologies, Vancouver, Canada.

**Chemicals and Reagents:** All reagents and antibodies were purchased from the following: Sigma-Aldrich, St. Louis, MO, USA (Celastrol-C0869); Thermo Scientific, Waltham, MA, USA (RPMI-1640-SH30027), Corning Life Sciences, USA (Penicillin/Streptomycin-30-002-CI); InvivoGen**,** San Diego, CA, USA (Puromycin-58-58-2); STEMCELL Technologies, Vancouver, BC, Canada (MethoCult-H4100 -04100); Molecular Probes, Eugene, OR, USA (TO-PRO-3).Cell Signaling Technology, Beverly, MA, USA (NPM1-ALK-3333, pNPM1-ALK-12127, pSTAT3-9145), pAKT-4060, AKT-2920, ERK1/2-4695), Survivin-2802, C-Myc-5605); BD Biosciences San Jose, CA, USA (β-Actin-612656, STAT3-610189, pERK1/2- 612358, PARP-556949, Caspase 9-551246), Caspase 8-551242, BAX-610982, FITC-CD30 (555829), FITC-IgG1κ isotype control-555748, FITC-Annexin V-556419; Sigma-Aldrich (FLAG-F3165, BCL2-B9804)

**Cloning and generation of stable Ba/F3-FG-NPM1-ALK cell line:** Human full-length NPM1-ALK cDNA (a gift from Dr. Toshiki Watanabe, The University of Tokyo, Japan) was amplified by PCR. The PCR product was then gel purified, digested with XbaI and NheI restriction enzymes and subcloned into lentiviral plasmid pCDH-EF1-MCS-T2A-Puro vector (purchased from System Biosciences, Palo Alto, CA, USA). The colonies were screened and positive clones were confirmed by Sanger DNA sequencing.

The Lentiviral plasmids, either empty vector pCDH-EF1-MCS-T2A-Puro or recombinant plasmid pCDH-EF1-FG-NPM1-ALK, were transfected along with packaging and envelop plasmids psPAX2, and pMD2.G into HEK293T cells. The lentiviral particles were collected after 48 hours. The Ba/F3 cells were transduced with either empty vector or pCDH-EF1-FG-NPM1-ALK lentivirus, and selection was carried out after 48 hours with 2 µg/ml puromycin. The Ba/F3-pCDH-vector cell growth was IL3-dependent and transformed Ba/F3-FG-NPM1-ALK cells became IL3-independent. The fusion gene NPM1-ALK mRNA expression levels were confirmed by qPCR and protein levels were confirmed by western blotting using Flag and NPM1-ALK antibodies.

**Flow Cytometry:** Apoptosis of untreated and treated cells (including normal T cells) was analyzed by flow cytometry with FITC-annexin V and TO-PRO-3 staining. The cells were treated with celastrol from 0.25 to 1 μM for 48 hours. After the incubation period, cells were harvested, washed with PBS, and incubated with FITC-Annexin and TO-PRO-3 diluted in Annexin V binding buffer for 15 minutes at room temperature. The analysis was performed using BD Accuri C6 plus flow cytometer (BD Biosciences San Jose, CA, USA).

**Western blot analysis**: Control and treated cells were harvested after the designated period and washed with PBS. The cells were then lysed in lysis buffer (25mM Tris. HCl, 150mM NaCl, 25mM NaF, 0.5mM Na-orthovanadate, 1% Triton-X, 1mM Benzamidine) with protease and phosphatase inhibitors. The pellets were incubated with lysis buffer on ice for 20 minutes, centrifuged at 10,000 RPM for 15 minutes and protein concentrations were determined using BCA protein assay kit (Pierce Biotechnology, Rockford, IL USA). The protein samples were separated by SDS-PAGE, immunoblotting was performed, and blots were scanned using the Odyssey IR scanner (Li-cor Biosciences, Lincoln, NE, USA).

**Clonogenic assay:** The clonogenic assay was performed to determine the effect of celastrol treatment on clonogenic survival of NPM1-ALK-positive cell lines Karpas-299 and SUP-M2. The cells were treated with 0.5 - 1.0 μM of celastrol. After incubation for 24 hours, the cells were diluted to 1000 cells with MethoCult medium and plated in triplicates. The cells were allowed to grow and form colonies for 8 days. Celastrol treated plates showed colony inhibitory effect when compared to the control plate. The colonies were scanned and counted using the Nexcelom Celigo imaging system.

**Analysis of CD30 expression:** The SUDHL-1 and Karpas-299 cell lines were treated with 0.5 - 1.0 µM concentration of celastrol for 24 hours. The cells were harvested and washed with PBS and incubated with antibodies FITC-conjugated CD30 antibody or FITC-IgG1κ isotype control diluted in 0.2% BSA/PBS on ice for 1 hour. After the incubation period, cells were washed with 0.2% BSA/PBS and the pellet was resuspended in 200 µl of 0.2% BSA/PBS and analyzed using flow cytometry.

**Statistical analyses:** One-way ANOVA analysis with Dunnett’s test method (SigmaPlot, version 13.0; Systat Software) was performed to determine significant differences in values between treated and untreated ALCL cells under different experimental conditions. Significance was defined as p- values < 0.05.
